# Dietary Strategies to Reduce Obesity Burden—Polyphenols as a Game-Changer?

**DOI:** 10.3390/healthcare10122430

**Published:** 2022-12-02

**Authors:** Anat Yaskolka Meir, Gal Tsaban

**Affiliations:** 1 Department of Epidemiology, Harvard T.H. Chan School of Public Health, Boston, MA 02115, USA; 2 Division of Cardiology, Soroka University Medical Center, Beer-Sheva 84101, Israel

The obesity epidemic has nearly tripled worldwide over the past five decades and has become a significant risk factor for noncommunicable diseases [1]. The burden of obesity extends beyond excess weight, as individuals with obesity are at a higher risk of mortality and significant morbidities such as diabetes mellitus, hypertension, dyslipidemia, hepatosteatosis, and sleep apnea, which can eventually lead to an increased cardiovascular and cancer risk [2]. These obesity-related conditions affect the patient both physically and mentally.

Obesity also has a sizeable economic impact beyond the individual’s physiological and psychological health. Direct economic implications can be dichotomized into medical or nonmedical [3]; the indirect costs, on the other hand, are mostly related to the loss of productivity (morbidity and mortality costs). These considerations also promote obesity-related disease treatments.

Over the years, researchers have struggled with the question of what is the best dietary approach to reduce the obesity burden and its subsequent health outcomes. Furthermore, which dietary changes will lead to long-lasting health, even beyond weight loss. Modern nutritional research has been extended beyond general dietary concepts, testing the effects of specifically added dietary components such as polyunsaturated fatty acids (PUFAs), dietary fibers, polyphenols, and dietary modifications such as reducing red and processed meat. The challenges in generalizing dietary interventions are caused by several factors: (i) the “live-laboratory” monitoring in these studies does not apply to real-life settings; (ii) interventions that are impossible to replicate (for example, providing the participants with agents and supplements not available to the public); and (iii) the long-term effect post-intervention of the diets is not clear.

The Mediterranean diet is one of the most rigorously studied dietary approaches. It constitutes an eating pattern that varies between different Mediterranean countries and is based on consuming extra virgin olive oil; a high consumption of legumes and nuts, unrefined cereals, fruits, and vegetables; a moderate to high consumption of fish; a moderate consumption of dairy products (mainly, cheese and yogurt) and wine; and a low consumption of meat and meat products [4]. The advantages of the Mediterranean diet in promoting health and reducing the obesity burden are well established and include a cardiovascular risk reduction [5,6], lower all-cause mortality [7], an improved glycemic response [6], and reduced hepatosteatosis and nonalcoholic fatty liver disease (NAFLD) prevalence [8].

Two landmark dietary intervention studies that included the Mediterranean diet were published, emphasizing the beneficial effect of the Mediterranean diet on obesity-related diseases. In the weight-loss-directed 2-year Dietary Intervention Randomized Controlled Trial (DIRECT) [6] conducted over 15 years ago, 322 participants were randomly assigned to low-fat (LF), Mediterranean, and low-carbohydrate (LC) diets. In this trial, the Mediterranean diet was found to have a favorable cardiometabolic benefit that was also sustained four years after the intervention’s end despite substantial weight regain [9]. The Prevención con Dieta Mediterránea (PREDIMED) trial [5] enrolled 7447 men and women with a high cardiovascular risk, but with no cardiovascular disease at enrollment, to eat either a Mediterranean diet supplemented with extra virgin olive oil, a Mediterranean diet supplemented with mixed nuts, or a control diet (with the advice to reduce dietary fat). Assignment to an energy-unrestricted Mediterranean diet supplemented with either extra virgin olive oil or nuts was associated with a lower risk of major cardiovascular events over five years compared to the assignment to a control (LF) diet.

The beneficial effect of the Mediterranean diet is attributed to the metabolomic profiles of individual nutrients, such as polyphenols. The polyphenol content in the Mediterranean diet is relatively high and attributed to the high content of plant food sources. The mean polyphenol intake in the traditional Spanish Mediterranean diet was estimated to be between ~2500 and 3000 mg/day [10] compared to ~1000 mg/day in a Western-style diet [11].

Polyphenols are secondary metabolites of plants with antioxidant properties [12]. The main groups of polyphenols are classified by the number of phenol rings they contain and by the structural elements, including phenolic acids, flavonoids, stilbenes, and lignans. There are several types of polyphenols in food, and edible products may contain a mixture of different polyphenols [13]. Polyphenol content is affected by environmental factors (such as soil type or exposure to light), storage conditions, and different methods of cooking [13]. Among the 100 richest dietary sources of polyphenols are cloves, cocoa powder, black olives, green tea, extra virgin olive oil, different berries and nuts, and wine [14]. A small portion (5–10%) of polyphenols and their metabolites are absorbed in the human body, depending on their degree of structural complexity and polymerization [15].

Several short-term interventions that included polyphenol enrichment were performed in an overweight or obese population to examine their effect on weight loss and obesity-related cardiometabolic diseases. After 12 weeks of oral resveratrol (a polyphenol of the stilbenoid group) supplementation in obese patients, total cholesterol and HDL cholesterol were improved [16]. A 2-month dietary supplement based on a combination of Lippia citriodora and Hibiscus sabdariffa polyphenolic extracts in overweight patients led to a remarkable improvement in body weight, triceps skinfold thickness, body fat, and hip circumference compared to a placebo group [17]. Additionally, differences in heart rate and blood pressure parameters between the group taking the supplement and the placebo group were observed. A double-blind, randomized, controlled trial was performed among 35 adults with obesity to examine the effect of polyphenol-rich juçara fruit supplementation on metabolic parameters [18]. Following six weeks of either a placebo or 5 g of lyophilized juçara supplementation, the supplementation with juçara effectively reduced body fat and increased HDL cholesterol. An 8-week, low-calorie, high-polyphenol cranberry beverage reduced serum insulin and improved HDL cholesterol in healthy overweight or obese patients [19].

In the 24-month-long CardiovaSCulAr Diabetes & Ethanol (CASCADE) trial [20], 224 participants were randomly assigned to mineral water, white wine, or red wine intervention groups, and it was found that moderate wine intake, especially of red wine known to be rich in polyphenols, among well-controlled diabetics as part of a healthy diet modestly decreases cardiometabolic risk. A 16-week-long, randomized, controlled intervention in 72 overweight and obese but otherwise healthy individuals aimed at studying the effects of a polyphenol-rich flavonoid supplementation in addition to the Mediterranean diet on health-related quality of life [21]. Compared to a placebo + Mediterranean group, the supplementation + Mediterranean diet had a greater DEXA-measured body fat mass decrease, mainly within the trunk area. Recently, we have shown that a polyphenol-enriched Mediterranean diet had a beneficial effect on the cardiometabolic risk parameters after six months [22], and overall, at 18 months, weight loss and waist circumference reductions were higher for those partaking in the Mediterranean and polyphenol-enriched Mediterranean diets [23].

In this Special Issue of *Healthcare* titled, “Obesity and its related complications—current treatments and future aspects”, we aim to provide an update on the different aspects of obesity, its related cardiometabolic diseases, and current and future prevention strategies and treatments. We also aim to spotlight strategies to reduce the physiological, emotional, sociological, and economic burden of obesity and cardiometabolic diseases.
